# Occurrence of Femoral Nerve Palsy After Total Hip Arthroplasty (THA) Using the Direct Lateral Approach

**DOI:** 10.7759/cureus.74636

**Published:** 2024-11-27

**Authors:** Yasmin Nached, Zeinab Al-Rawi, Abdulla Abdelwahab, Ahmed Elsayed, Ali H Ismaeil

**Affiliations:** 1 Department of Orthopaedics and Trauma, Mohammed Bin Rashid University of Medicine and Health Sciences, Dubai Health, Dubai, ARE; 2 Department of Orthopaedics and Trauma, Mohammed Bin Rashid University of Medicine and Health Sciences, Dubai, ARE

**Keywords:** electromyography, femoral nerve palsy, microscopic polyangiitis, nerve conduction studies, total hip replacement

## Abstract

Femoral nerve palsy (FNP) is a rare but serious complication after total hip replacement (THP). Despite its rarity, FNP can significantly impact patient recovery and quality of life. This case report examines the occurrence of FNP in a patient following a primary THP and highlights the importance of surgical technique and postoperative detection and its management. We present the case of a 38-year-old male with a history of microscopic polyangiitis on long-term steroid treatment, who developed FNP following THP. The patient was admitted with non-traumatic right hip pain with osteoporotic fracture of the femoral head and underwent elective THP. Postoperatively, the patient showed quadriceps weakness and related sensory deficits. Postoperative assessments included physical examination, electromyography (EMG), nerve conduction studies (NCS), and magnetic resonance imaging to assess the extent of the nerve injury. EMG and NCS confirmed severe femoral mononeuropathy with profound active denervation changes. A subsequent magnetic resonance imaging revealed atrophy of the right sartorius and quadriceps femoris muscles. Conservative management was decided, including physiotherapy and close follow-up, which led to significant gradual improvement over six months, with enhanced knee range of motion (ROM), increased quadriceps strength, and improved sensation on the medial side of the leg and foot. Femoral nerve injuries, although uncommon, pose significant risks in THP. Excessive retraction during surgery may contribute to these injuries. Early diagnosis, conservative management, and interdisciplinary coordination are crucial to achieve optimal recovery.

## Introduction

Total hip replacement (THR) is a well-recognized surgery that addresses hip pain, functional limitations, and stiffness arising from degenerative diseases, avascular necrosis, trauma, and dysplasia, among many other conditions, yielding high levels of patient satisfaction by significantly improving the quality of life [1]. One of the most feared complications is femoral nerve palsy (FNP), which, despite being well-documented in the literature, remains rare, with an incidence ranging from 0.1% to 0.4% [2].

The femoral nerve, the largest branch of the lumbar plexus, originates within the psoas major muscle of the posterior abdominal wall and descends inferiorly deep to the mid-inguinal point, giving off a branch to the iliacus muscle along its course. The femoral triangle gives off a branch to the pectineus and then divides into anterior and posterior divisions. The anterior division gives a motor branch to the sartorius muscle and a cutaneous branch that innervates the anteromedial thigh, whereas the posterior division supplies the quadriceps femoris muscle and terminates distally as the saphenous nerve, which innervates the medial aspect of the leg and foot. Therefore, FNP can manifest with sensory and/or motor symptoms, such as hypoesthesia or painful paresthesia along the sensory distribution and/or quadriceps weakness, respectively [3].

In most cases, the origin of the palsy is unknown; however, the majority occur intra-operatively, with an incidence of 60% due to iatrogenic causes [4]. These causes can include direct trauma, hemorrhage, retraction, and leg lengthening [2,5,6]. The role of the surgical approach in nerve injury remains controversial; however, one study has shown that the incidence of FNP is 14.8 times higher with an anterior approach, including both the direct anterior (Smith-Peterson) and anterolateral (Watson-Jones) approaches [7]. At our institute, we prefer a lateral approach in a supine position to avoid the course of the femoral nerve. Several risk factors have been reported, such as the use of anticoagulants, female gender, and a history of hip dysplasia. There is no definitive protocol for addressing postoperative FNP, but conservative management with physiotherapy and/or bracing has shown satisfactory results [6].

We present a case of a 38-year-old male with a history of microscopic polyangiitis on long-term steroids for four years. He sustained FNP following a THP. In this paper, we underscore the risk factors for FNP including the surgical approach as well as discuss the recovery course, highlighting the role of conservative management.

## Case presentation

A 38-year-old male presented to our trauma center's emergency department (ED) with a complaint of non-traumatic right hip pain and limited range of motion (ROM) persisting for the last two months, which escalated in the past few days, hindering his ability to walk due to severe pain. The patient has a history of autoimmune disease and was on long-term steroid treatment until one year ago. He had previously undergone a THP on the left side and core decompression of the right hip one year from the presentation.

Upon examination, the patient was afebrile with a temperature measured at 36.7°C. The patient exhibited noticeable tenderness and reduced ROM in the right hip. No local signs of infection were observed, and the distal neurovascular status remained intact. 

Laboratory evaluation revealed a white blood cell (WBC) count of 12.3 (normal: 5.0-15 10^3^/uL), and a C-reactive protein (CRP) of 9.6 mg/L (normal: 0-5 mg/L).

**Figure 1:**
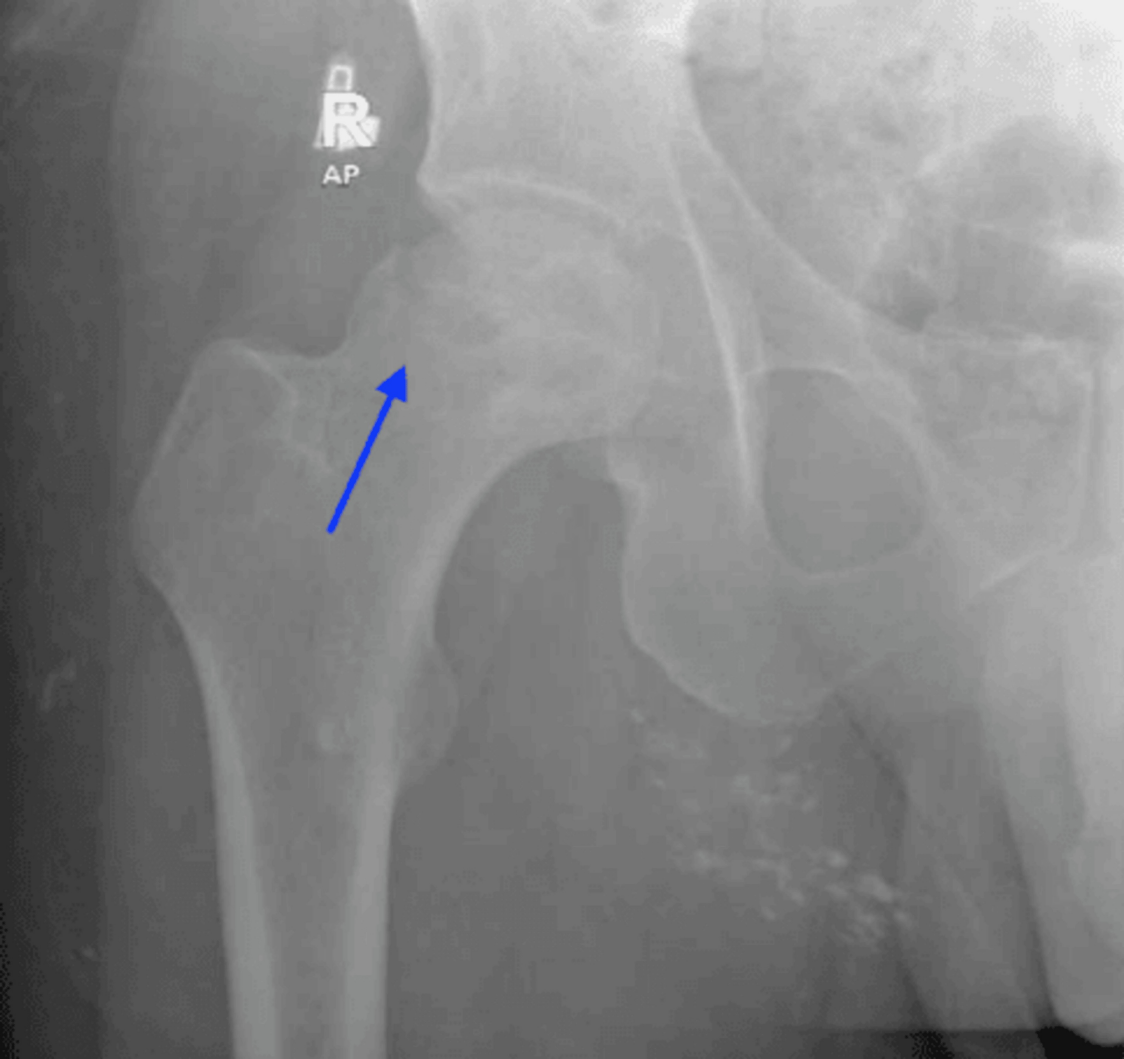
X-ray of the right hip showing focal cortical irregularities in the femoral head and neck junction

A plain radiograph of the right hip revealed focal cortical irregularities and lucency in the superior lateral portion of the femoral head and neck junction, suggestive of a likely osteoporotic fracture (Figure 1).

**Figure 2:**
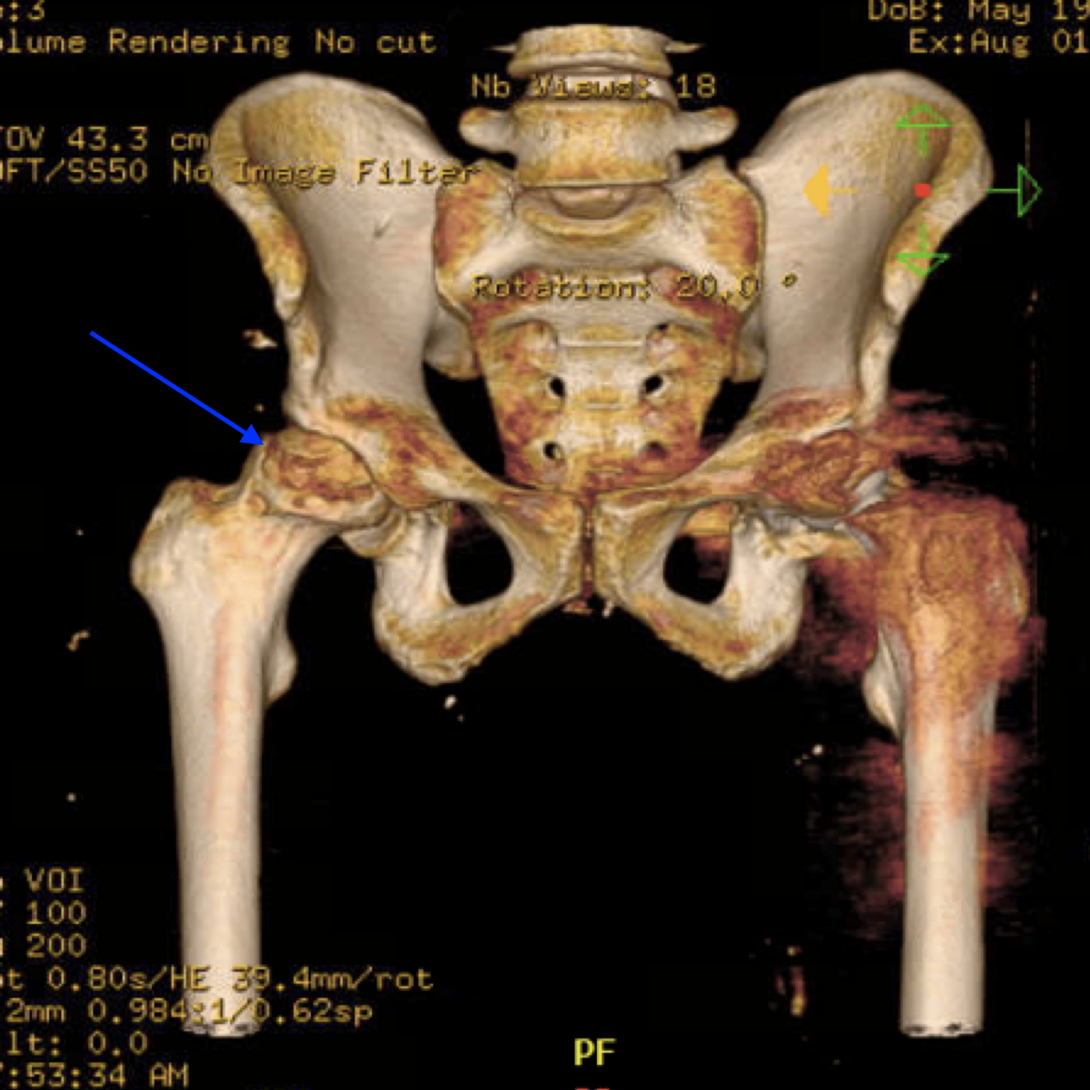
CT scan showing osteolytic lesions in the right femoral head

To conduct a more thorough evaluation, a CT scan (Figure 2) was requested, revealing multiple osteolytic lesions in the right femoral head, indicative of what appears to be several pathological comminuted fractures.

**Figure 3:**
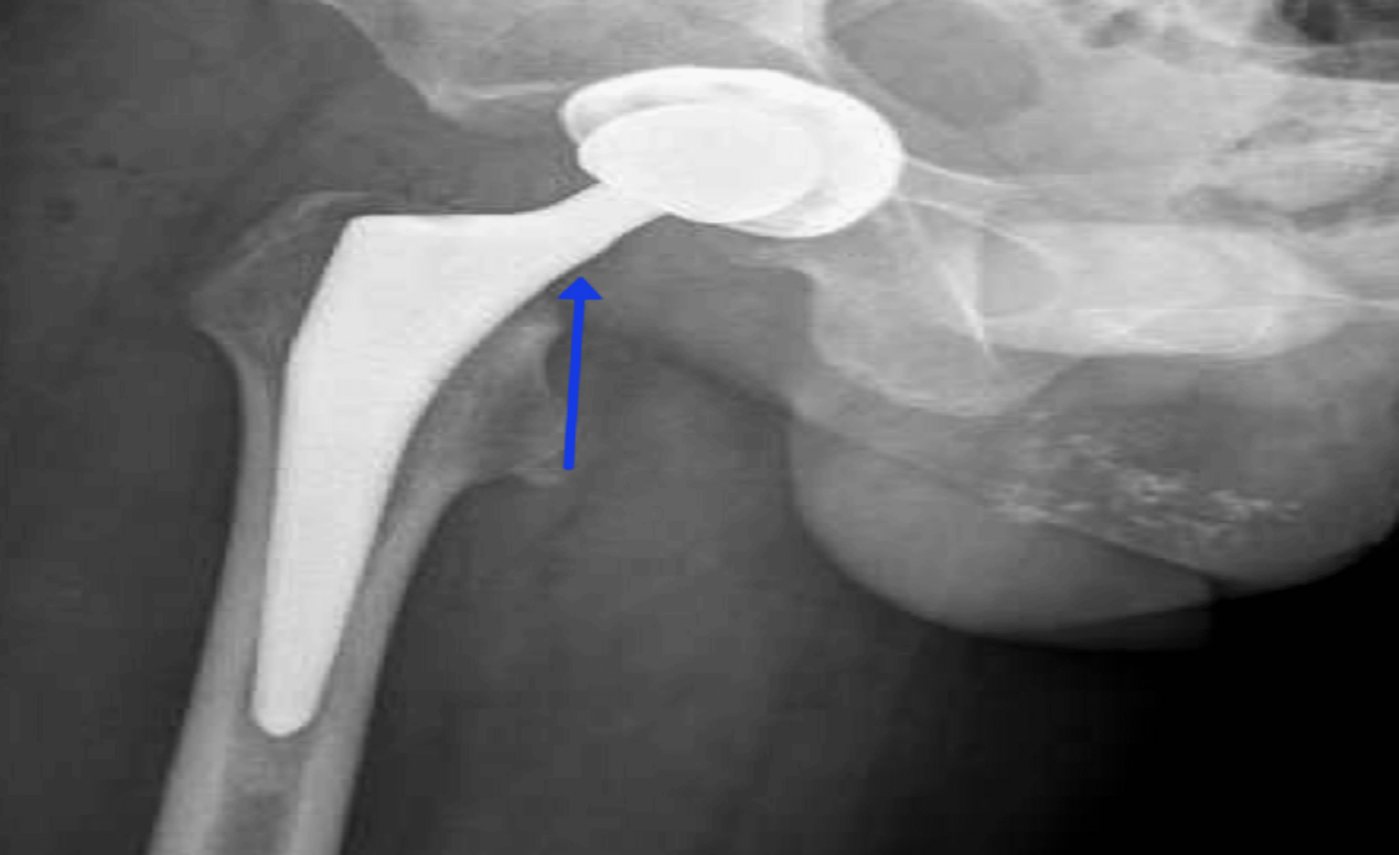
Postoperative X-ray showing good positioning of the implant

The patient was released with a pain management plan and advised to limit weight-bearing as tolerated. A THR surgery for the right hip was scheduled electively as the patient’s pain became intolerable and is affecting his daily activity. The patient was admitted and surgery was performed without any unexpected intra-operative events. Postoperatively, the patient underwent physiotherapy rehabilitation and daily wound dressing without any complications. The inpatient recovery period revealed postoperative weakness of the quadriceps muscles and sensory deficit of the medial side of the leg and foot. Postoperative X-ray images were taken (Figure 3), showing a well-accepted position of the implant. The patient was reviewed by the arthroplasty team, who decided on conservative treatment. The plan was discussed with and explained to the patient prior to discharge. He was discharged once he was fit and was scheduled for an outpatient department (OPD) visit four weeks later.

Upon discharge from the hospital, the patient was instructed to continue physiotherapy rehabilitation with full weight-bearing (FWB) as tolerated, emphasizing adherence to full ROM mobilization. However, during his first OPD, the patient, utilizing a walker with knee support, raised concerns about a potential femoral nerve injury. Upon examination, flaccidity in the quadriceps muscles and noticeable weakness in hip flexion were observed. The patient faced difficulties in knee extension, and the absence of the knee jerk reflex was noted. Sensory deficits to light touch on the medial aspect of the calf and foot were also identified.

**Figure 4:**
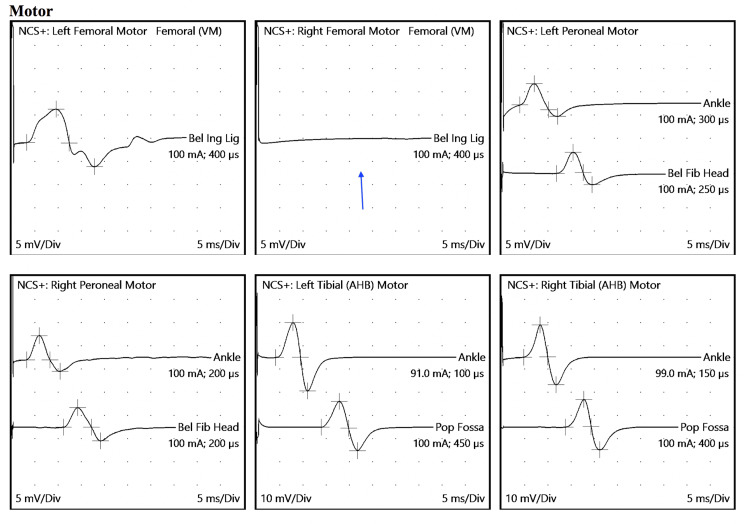
NCS of right femoral nerve showing no recordable response NCS, nerve conduction study

Subsequently, one month after surgery, electromyography (EMG) and nerve conduction study (NCS) tests were conducted. The EMG revealed no recordable amplitudes for the right femoral and right saphenous nerves, indicating profound active denervation changes in muscles innervated by the right femoral nerve. This suggested a severe, axonal femoral mononeuropathy proximal to the branch of the iliopsoas muscle (Figure 4). NCS exhibited motor involvement with no recordable response in the right femoral nerve and sensory involvement with no recordable response in the right saphenous sensory nerve.

**Figure 5:**
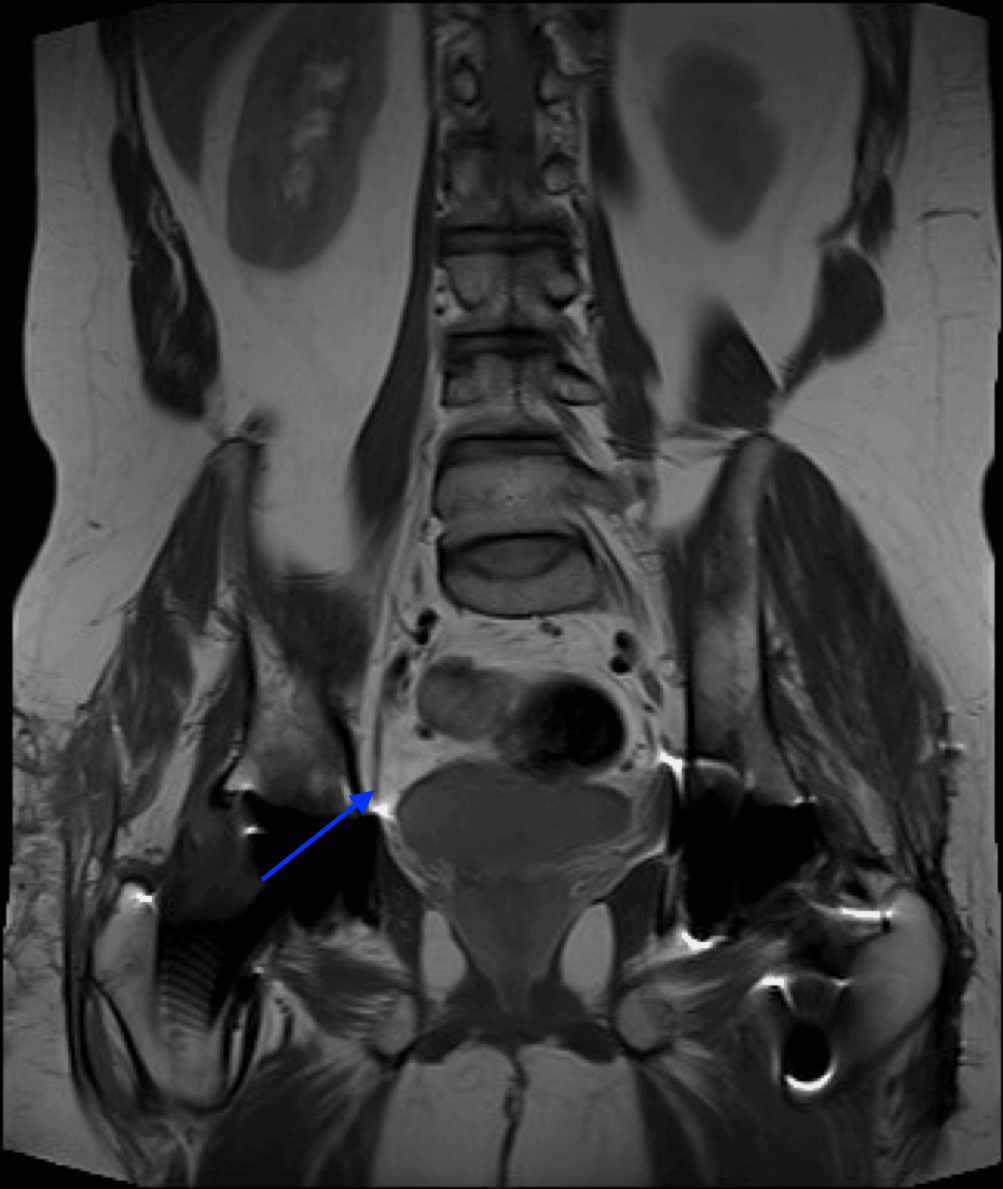
Magnetic resonance imaging showing an intact right femoral nerve along its course into the thigh

**Figure 6:**
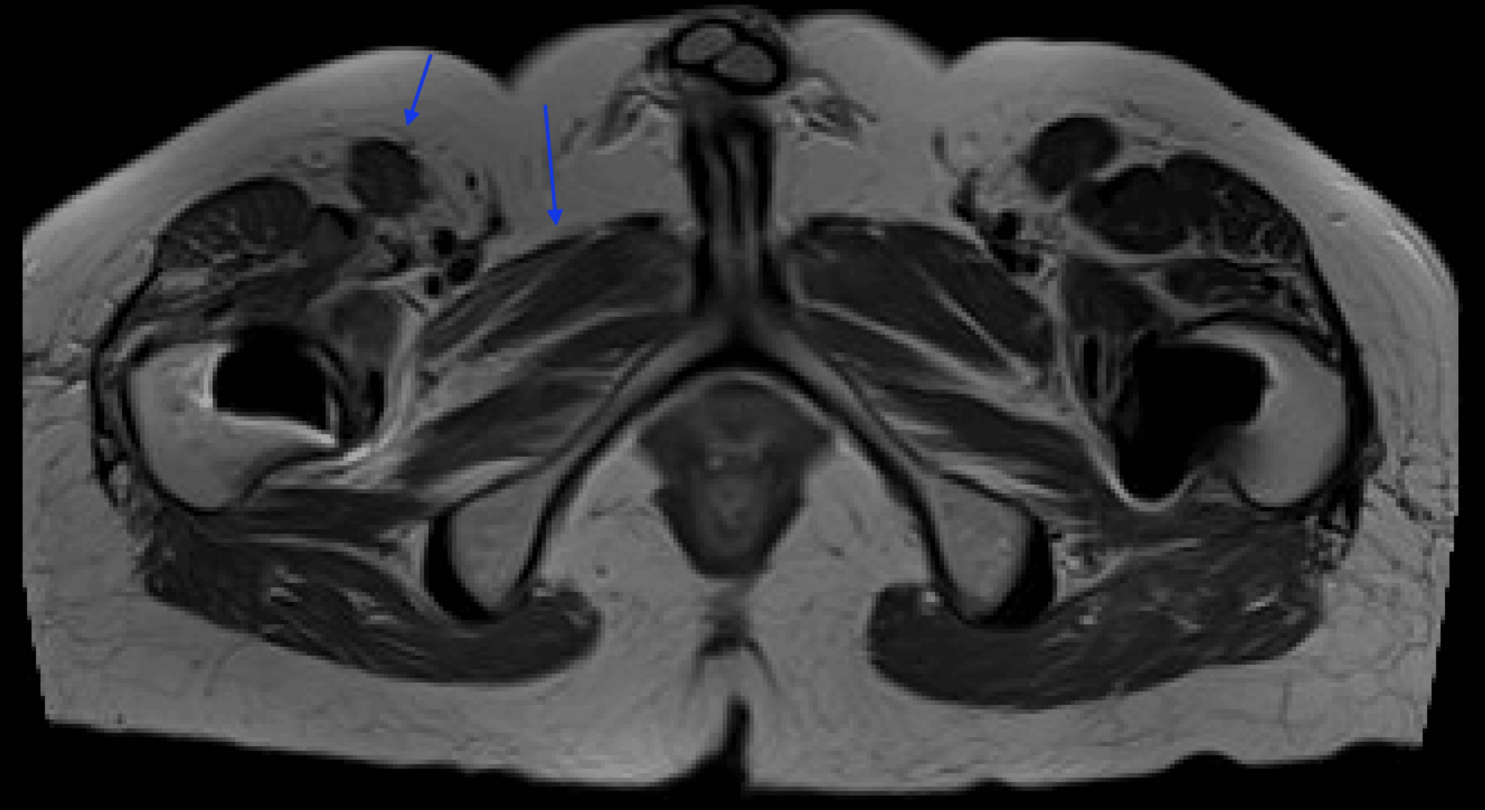
Magnetic resonance imaging showing the atrophied muscles

A month later, a magnetic resonance imaging of the right thigh (Figure 5) was performed, revealing the right femoral nerve along its course into the thigh without any notable thickening or discontinuity. The imaging displayed moderate to severe atrophy of the right sartorius and quadriceps femoris, with mild atrophy of the adductor muscles (Figure 6).

Two months later, a repeat of the EMG and NCS tests indicated no alteration in sensory or motor potential on NCS. However, the EMG revealed mild re-innervation changes in the iliopsoas muscle, manifested by the presence of some broad polyphasic motor unit potentials, which were not observed in the previous assessment (Table 1).

**Table 1:** Needle EMG results showing active degenerative changes of right vastus medialis and iliopsoas, in addition to showing broad polyphasic units suggestive of reinnervation of iliopsoas muscle EMG, electromyography

Side	Muscle	Ins Act	Fibs	PSW	Fasciculation	Others	Amplitude	Duration	Poly	Recruitment	Int Pattern
Right	Tib Anterior	N	-	-	-	-	No MUAPs				
Right	Vastus Med	N	2+	2+	-	-	Nml	Nml	-	Nml	N
Right	Iliopsoas	N	1+	1+	-	-	N/H	Broad	1+	R50%	50%
Right	Gluteus Med	N	-	-	-	-	Nml	Nml	-	Nml	N

Continuing follow-up in the OPD, the patient was advised to use a knee immobilizer to maintain knee extension and engage in physiotherapy strengthening exercises for the right thigh muscles. A plan for another set of EMG and NCS tests in three months was established.

Upon the subsequent visit, after six months of surgery, the patient displayed remarkable improvement. There was evident enhancement in knee ROM and an increase in the strength of the right quadriceps. The patient had successfully achieved a full extension of the knee, and there was a significant improvement in sensation on the medial side of the leg and foot.

## Discussion

Femoral nerve injuries, though uncommon, are a significant concern in THP surgeries. Up to 60% of these injuries are iatrogenic in nature, underscoring the importance of identifying and mitigating risk factors during surgical procedures [8].

Direct nerve injury can arise from intra-operative positioning, aggressive surgical approaches, mistaken anatomy, diathermy burns, power tools, and nerve entrapments within fractures during reduction [9,10]. In THP surgeries using a direct lateral approach, precise placement of retractors is crucial to minimize risks such as femoral nerve injuries. The anterior retractor is positioned carefully to avoid damage to the femoral neurovascular bundle, and it is aided by a Cobb elevator to establish a safe surgical plane between the anterior acetabular wall and the capsule. Simultaneously, a superior retractor protects and elevates the gluteus muscle, while a posterior Mueller retractor shields against the ischium. These techniques not only facilitate safe tissue excision and acetabular reaming but also address anatomical variations, reducing potential nerve injury risks [11]. Additionally, systematic reviews emphasize the importance of considering factors like leg lengthening. Acute limb-lengthening of more than 2-4 cm during arthroplasty has been associated with an increased risk of neural injury, highlighting the need to minimize leg length discrepancies through careful preoperative planning and precise measurement techniques to enhance surgical safety and optimize patient outcomes [6,12].

Additionally, conditions such as anticoagulant therapies and coagulopathies can cause spontaneous bleeding in the soft tissues adjacent to the nerve, potentially leading to hematoma formation compressing the adjacent nerve or a temporary loss of view increasing the risks of nerve injuries [6].

In this case, the femoral nerve injury following THP using the direct lateral approach is particularly notable given its rarity, with femoral nerve injuries generally occurring in only 0.0% to 2.3% of cases. This approach is more commonly associated with superior gluteal nerve injury, which occurs in 2.2% to 42.5% of patients [13-18].

While retractors are typically used to protect neurovascular structures during surgery, improper placement can inadvertently cause nerve damage by either crushing the nerve or positioning it too close to the saw blade or drill, thereby increasing the risk of injury.

The application of retractors over the anterior acetabular rim, although intended to provide better exposure, may have contributed to nerve stretching and subsequent injury. Excessive retraction by the resident or assistant scrubbed in to improve their view could have exacerbated this issue, leading to further complications.

Our findings highlight the necessity for surgeons to be cognizant of potential complications associated with different surgical approaches. The lateral approach in the supine position, while advantageous in certain aspects, requires careful consideration of anatomical and technical factors to minimize the risk of femoral nerve injury. This type of injury is categorized as a type 1 iatrogenic peripheral nerve injury, indicating that the nerve injured was not the target of the treatment [19].

Diagnosing femoral nerve injury after total hip arthroplasty requires a comprehensive approach, involving imaging and neurophysiological studies, following careful clinical evaluation and assessment. Imaging techniques assess fixation adequacy and metalwork issues, with MRI specifically revealing nerve characteristics like thickening and continuity of the nerve indicative of injury, in addition to the muscle status. Neurophysiological studies such as EMG and NCS play a critical role in localizing and grading the nerve injury, aiding in treatment planning and monitoring recovery [13]. EMG is typically best performed six to eight weeks after surgery to ensure accurate results after Wallerian degeneration has progressed sufficiently. Early EMG soon after injury may not always indicate the full extent of nerve damage [9,20]. Clinical signs such as neuropathic pain complement these studies, offering valuable insights into nerve recovery progression and guiding timely interventions. Optimal management of these injuries depends on coordinated efforts among orthopedic surgeons, neurologists, and rehabilitation specialists, ensuring tailored treatment plans that optimize patient recovery outcomes [6].

The management of such injuries remains conservative, as there is no established protocol in the literature for managing them [7,13]. Conservative management includes physiotherapy with joint mobilization, extended bracing, and expectant waiting for a functional return. Recovery periods in the literature are noted to be typically six months to less than two years, with the potential for full recovery without motor deficits or long-term disability [7].

Further research is warranted to explore the comparative outcomes and complication rates between various surgical approaches to refine practices and enhance patient safety. Early recognition and interdisciplinary cooperation in managing femoral nerve injuries post-THP are crucial. Recovery can vary based on the severity and location of the injuries, but the potential for continued improvement over time should be communicated to patients. It is essential for healthcare providers to discuss realistic expectations with patients regarding their recovery and long-term outcomes.

## Conclusions

Femoral nerve injuries are a serious complication in THP surgeries, with several identifiable risk factors contributing to their occurrence. This case report emphasizes the need for careful surgical planning and technique, particularly when employing approaches such as the direct lateral approach, which is a very common surgical approach used worldwide for total hip arthroplasty. Early recognition and comprehensive management of femoral nerve injuries can significantly improve patient outcomes, highlighting the importance of ongoing research and surgical refinement in this field.

## References

[1] Learmonth ID, Young C, Rorabeck C (2007). The operation of the century: total hip replacement. Lancet.

[2] Schmalzried TP, Amstutz HC, Dorey FJ (1991). Nerve palsy associated with total hip replacement. Risk factors and prognosis. J Bone Joint Surg Am.

[3] Moore KL, Dalley AF, Agur AMR (2013). Clinically Oriented Anatomy. Clinically Oriented Anatomy 7th edn.

[4] Kretschmer T, Heinen CW, Antoniadis G, Richter HP, König RW (2009). Iatrogenic nerve injuries. Neurosurg Clin N Am.

[5] McConaghie FA, Payne AP, Kinninmonth AW (2014). The role of retraction in direct nerve injury in total hip replacement: an anatomical study. Bone Joint Res.

[6] Fox AJ, Bedi A, Wanivenhaus F, Sculco TP, Fox JS (2012). Femoral neuropathy following total hip arthroplasty: review and management guidelines. Acta Orthop Belg.

[7] Fleischman AN, Rothman RH, Parvizi J (2018). Femoral nerve palsy following total hip arthroplasty: incidence and course of recovery. J Arthroplasty.

[8] Mehta CR, Constantinidis A, Farhat M, Suthersan M, Graham E, Kanawati A (2018). The distance of the femoral neurovascular bundle from the hip joint: an intraoperative guide to reduce iatrogenic injury. J Orthop Surg Res.

[9] British Orthopaedic Association Trauma Committee (2020). British Orthopaedic Association's Standards for Trauma (BOAST): Care of the older or frail patient with orthopaedic injuries. Injury.

[10] Yim GH, Lin Z, Shirley CP, Isherwood P, Power DM (2019). The late diagnosis of nerve injuries following interscalene block and shoulder surgery. J Musculoskelet Surg.

[11] Moretti VM, Post ZD (2017). Surgical approaches for total hip arthroplasty. Indian J Orthop.

[12] Johanson NA, Pellicci Pellicci (1983). Nerve injury in total hip arthroplasty. Clin Orthop Relat Res.

[13] Akinleye SD, Garofolo-Gonzalez G, Culbertson MD, Choueka J (2019). Iatrogenic injuries in percutaneous pinning techniques for fifth metacarpal neck fractures. Hand.

[14] Jacobs LG, Buxton RA (1989). The course of the superior gluteal nerve in the lateral approach to the hip. J Bone Jt Surg.

[15] Khan T, Knowles D (2007). Damage to the superior gluteal nerve during the direct lateral approach to the hip: a cadaveric study. J Arthroplasty.

[16] Picado CH, Garcia FL, Marques W Jr (2007). Damage to the superior gluteal nerve after direct lateral approach to the hip. Clin Orthop Relat Res.

[17] Oldenburg M, Müller RT (1997). The frequency, prognosis and significance of nerve injuries in total hip arthroplasty. Int Orthop.

[18] Ramesh M, O'Byrne JM, McCarthy N, Jarvis A, Mahalingham K, Cashman WF (1996). Damage to the superior gluteal nerve after the Hardinge approach to the hip. J Bone Joint Surg Br.

[19] Carter J, Pisquiy J, Polmear M, Khalifa R, Gonzalez G (2020). A new classification of iatrogenic peripheral nerve injuries. Biol Med.

[20] Pulos N, Shin EH, Spinner RJ, Shin AY (2019). Management of iatrogenic nerve injuries. J Am Acad Orthop Surg.

